# Contrast-enhanced magnetic resonance imaging (MRI) features of Gruberi Bursitis as a very rare cause of dorsolateral ankle pain and swelling: Case report and review of the literature^☆^

**DOI:** 10.1016/j.radcr.2022.04.061

**Published:** 2022-05-29

**Authors:** Yasser Ragab, Yasser Emad, Mariam Ahmed Saad, Johannes J. Rasker

**Affiliations:** a Radiology Department, Faculty of Medicine, Cairo University, Kasr Al-Ainy St, Cairo 11562, Egypt; b Rheumatology Department, Faculty of Medicine, Cairo University, Kasr Al-Ainy St, Cairo 11562, Egypt; c Medical Oncology Department, National Cancer Institute, Cairo, Egypt; d Faculty of Behavioral, Management and Social Sciences, Department Psychology, Health and Technology, University of Twente, Drienerloolaan 5, Enschede 7522NB, the Netherlands

**Keywords:** Gruberi sinus tarsi bursa, Gruberi bursitis, Enhanced MRI features of Gruberi bursitis

## Abstract

The Gruberi sinus tarsi bursa is a dorsolateral ankle anatomic bursa that has been described in the past but is rarely mentioned in recent radiology literature. The Gruberi bursa is distinguished by its position between the extensor digitorum longus tendons and the talus. It is usually unilocular, anechoic and compressible as shown with ultrasound in a previous study. In recent literature, the enhanced MRI features of an inflamed Gruberi bursa as the underlying cause of a painful ankle joint and antalgic gait are rarely demonstrated. In this report, we present the enhanced MRI features of Gruberi bursitis in a female patient who complained of acute onset of pain and swelling along the dorsolateral aspect of her left ankle, as well as a painful limping gait after sport-related activities. Complaints improved after an intra-bursal corticosteroid injection. The case is discussed and the typical enhanced MRI features are demonstrated. The relevant literature is discussed.

## Introduction

A synovial bursa is a small sac filled with synovial fluid, a liquid that acts as a cushion between muscles, tendons, and bones and helps to lubricate joints so that they can move freely. The Gruberi sinus tarsi bursa is an anatomic bursa located in the dorsolateral ankle, that is rarely mentioned in recent radiology literature. The Gruberi bursa is characteristically found between the extensor digitorum longus (EDL) tendons and the talus and 98% of the fluid collections identified on ultrasound (US) were unilocular, 94% were anechoic and 89% were found to be compressible, with the latter finding being more than coincidental [1].

In the current report, we present the characteristic enhanced MRI features of Gruberi bursitis in a female patient who complained of acute onset pain and swelling along the dorsolateral aspect of her left ankle, as well as a painful limping gait. The case is discussed, and the typical enhanced MRI features, as well as the relevant literature is reviewed.

## Case summary

A 32-year-old female patient presented to our facility with acute left ankle and foot pain along the dorsolateral aspect and a limping gait following sport-related activities, claiming a mild twisting ankle injury. Clinical ankle examination revealed normal anterior drawer test for anterior talofibular ligament integrity, inversion stress for calcaneofibular ligament integrity, and eversion stress tests for deltoid ligament integrity. Plain x-rays of the left ankle in anteroposterior (AP), mortise, and lateral views, as well as x-rays of the left foot in AP, lateral, and oblique views, revealed normal findings and ruled out a fissure fracture.

**Fig. 1:**
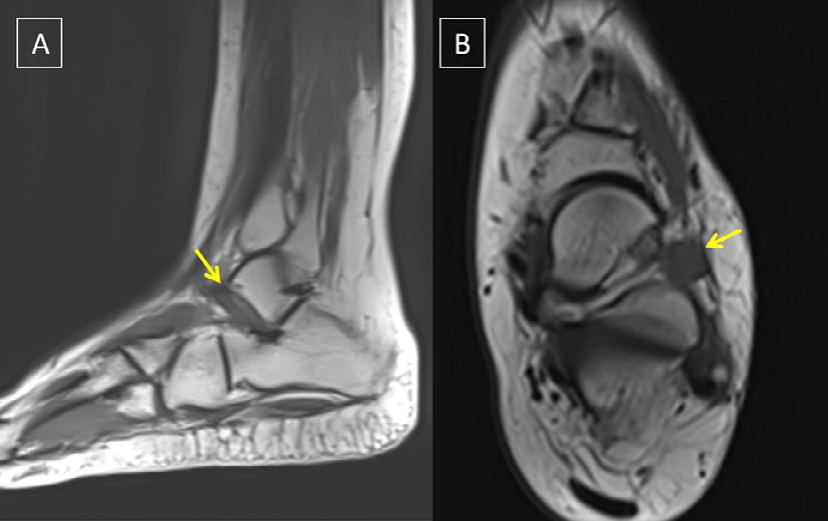
plain MRI, (A) sagittal, (B) axial showing mildly hypointense cystic collection at the dorsolateral aspect of the ankle region.

**Fig. 2:**
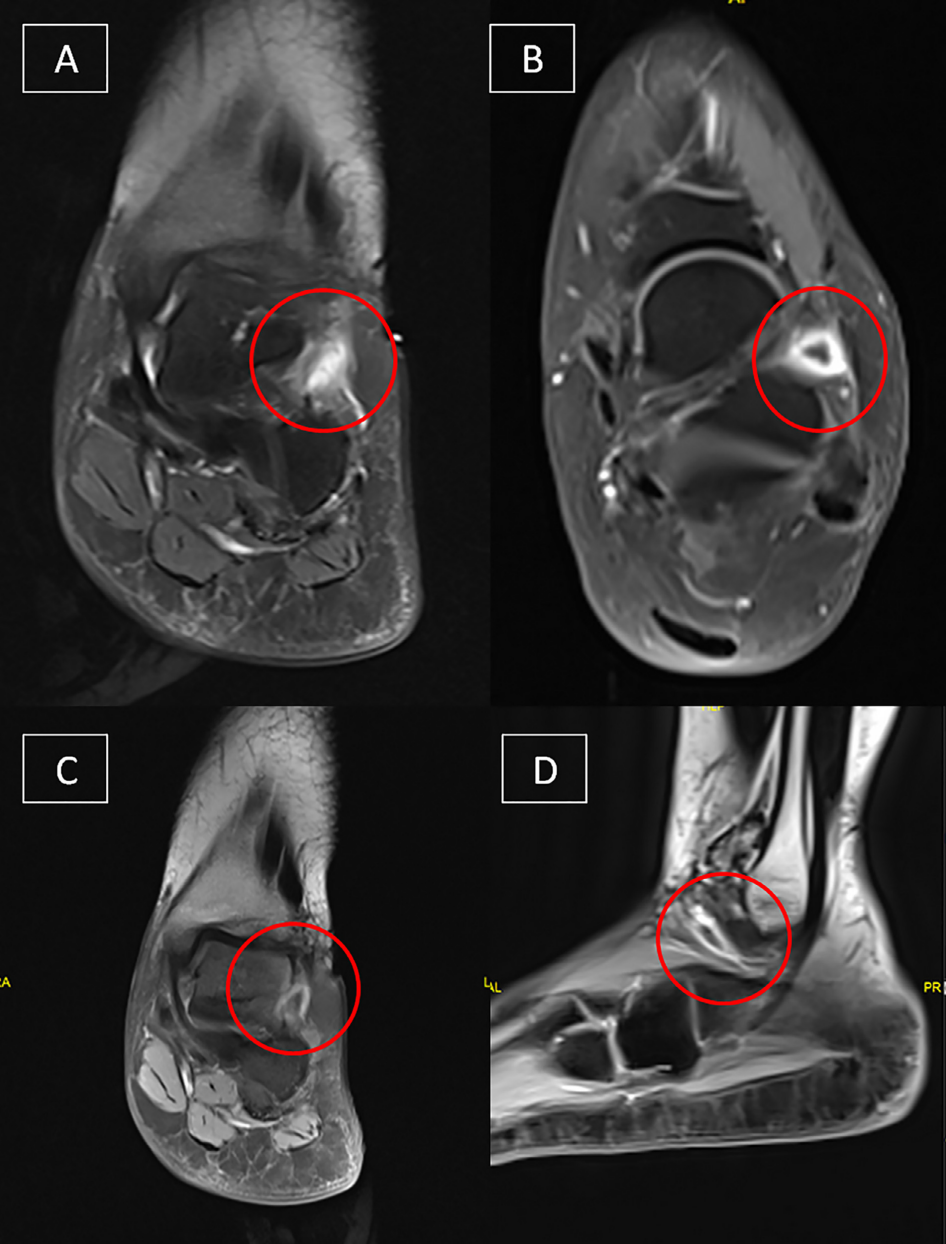
(A) axial and (B) coronal T1 FAT SAT with contrast demonstrating a well-defined ovoid cystic lesion (red circles) lining the inferior extensor retinaculum as it passes over the extensor digitorum longus tendon.(C) Coronal and (D) Sagittal images show the same findings (red circles). The precise anatomical locations of the latter, as well as the MRI findings, are consistent with a diagnosis of Gruberi bursitis. The bursa, on the other hand, is reaching the subtalar joint space but there is no definitive communication. Additionally, there is no associated ligamentous injury or osteochondral lesion, and the bone marrow signal intensity is normal.

The patient was treated conservatively with pain relievers, an ankle brace, and was advised to refrain from participating in sports for two weeks. Despite these measures, the patients' pain persisted, with little clinical improvement over the next two months following her initial presentation. A magnetic resonance imaging (MRI) of the patient's left ankle and foot was performed to rule out any underlying cause(s) of the patient's persistent pain despite early conservative measures and to rule out subclinical ligamentous injury, stress fracture, or osteochondral lesion. A mildly hypointense cystic collection was discovered on plain MRI at the dorsolateral aspect of the ankle (Fig. 1). While, the contrast-enhanced MRI revealed a well-defined ovoid cystic lesion lining the inferior extensor retinaculum as it passes over the extensor digitorum longus (EDL) tendon, with no communication with the ankle or subtalar joints (Fig. 2). The latter's precise anatomical location, as well as the MRI findings, are consistent with the diagnosis of Gruberi bursitis (Fig. 1). MRI also revealed normal bone marrow signal intensity, intact articular cartilage, and no associated ligamentous or osteochondral lesion. The fluid signal by MRI was not bright on T1, and together with the clinical presentation ruled out the possibility of infected bursitis, so diagnostic aspiration was not performed. Diagnosis of non-purulent inflamed Gruberi bursa was made, as evidenced by thickened marginal enhancement of the bursal boundaries An US guided injection of triamcinolone acetonide (Kenacort 40 mg) with 2 CC of added lidocaine was performed in the summit of the bursa. One week after the injection, the patient's ankle pain had improved significantly, and she was advised to continue wearing the ankle brace and ice packs at home, as well as avoid sports for a month. The clinical improvement seen in our patient supports the idea that the Gruberi bursa has an inflammatory component. The findings with contrast-enhanced MRI were essential to establish an appropriate diagnosis and take the best therapeutic decisions.

## Discussion

In the current case report we presented the MRI feature of Gruberi bursitis as a rare cause of dorsolateral ankle pain and swelling in a young female patient. Bursae around the foot and ankle can be anatomic, meaning they are present at birth at established anatomic sites, or adventitious, meaning they develop at sites of friction [2]. MRI and US examination tools are frequently used for additional evaluation of clinically suspected bursal inflammation, and abnormal fluid around the ankle on imaging may be a source of symptoms. Fluid can be seen in tendon sheaths and joints, as well as focal fluid in ganglion cysts and bursae [1].

The Gruberi sinus tarsi bursa is an anatomic bursa in the dorsolateral ankle that has been described but is rarely mentioned in the contemporary radiology literature [1]. The Gruberi bursa was first described by Alexander Monro (1773-1859) as "a bursa mucosa for the tendons of the extensor digitorum communis longus, between them and the tibia and ligament of the ankle" [3]. Similar references to this bursa can be found in several early twentieth-century anatomy textbooks, which defined the Gruberi sinus tarsi bursa's anatomical location between the dorsolateral aspect of the neck of the talus bone and the EDL tendon [3,4]. Furthermore, a potential communication between the bursa and the talonavicular joint has also been described [2], but none has been described between the bursa and the tibiotalar joint [1]. Jones [5] in 1949 claims that the bursa never communicates with the EDL tendon sheath, whereas a recent work by Kelikian and Sarrafian [6] stated that the bursa can communicate with the talonavicular joint, the tibiotalar joint, and the EDL tendon sheath. In our case, the precise anatomical locations, as well as the MRI findings, support a diagnosis of Gruberi bursitis. Although the bursa reaches the subtalar joint space, no definitive communication with the talonavicular joint, tibiotalar joint, or EDL tendon sheath can be established.

In a previous study, the Gruberi bursa was identified on US as a structure distinct from the tibiotalar and talonavicular joints. Furthermore, the Gruberi bursa was identified on US in 93% of ankles with a fluid collection at the dorsolateral ankle, and that the Gruberi bursa was unilocular, anechoic, and compressible, as well as being located between the EDL and the talus. Furthermore, a well-defined region between the EDL tendons and the dorsolateral talus was delineated after dissection of the ankle cadaveric specimen and injection of 2 mL of diluted blue latex (50% latex and 50% water), which anatomically corresponds to a Gruberi bursa [1]. With MRI the precise anatomical location of the Gruberi bursa was revealed in our case, with contrast enhancement indicating a non-infectious inflammatory process that was causing our patient's persistent ankle pain despite conservative treatment.

In a recent report, Weerakkody and Murphy [7] defined Gruberi bursa by MRI as the bursa that lines the inferior extensor retinaculum (frondiform ligament) as it passes over the EDL tendons at the anterior ankle joint. It is usually only visible on imaging if it is distended and can be mistaken for tenosynovitis, so it is also known as 'pseudo-tenosynovitis' of the EDL tendon. A Gruberi bursa will extend from the sinus tarsi and wrap around the EDL tendon, distinguishing it from fluid caused by tenosynovitis, which will extend along the tendon. Furthermore, the authors stated that on MRI, the fat surrounding the EDL tendon will be edematous and/or inflamed in tenosynovitis, and on both MRI and ultrasound, the fat surrounding the EDL tendon will be edematous and/or inflamed in tenosynovitis. In contrast to our findings, Weerakkody and Murphy [7] did not present post-contrast MRI images to document marginal enhancement, which clearly reflects an associated inflammatory component likewise in our case. Furthermore, our patient improved significantly after receiving a US-guided corticosteroid injection into the inflamed Gruberi bursa, in addition to other conservative treatments, confirming the presence of an inflammatory element that caused distension of this anatomical bursa and marginal enhancement as seen on post contrast MRI.

In a similar report, Roberts [8] presented the MRI findings of a distended bursa Gruberi in a 50-year-old male patient who had ankle pain that worsened when he exercised. The bursa Gruberi is distended. Furthermore the author mentioned that the EDL tendon is normal in thickness, with minor degenerative changes are evident at the point of contact between the anterior calcaneum and navicular, raising the possibility of a fibrous coalition.

## Conclusion

Gruberi bursa is by classification an anatomic bursa that is located in the dorsolateral ankle between EDL tendons and the dorsolateral talus. There is still debate about whether the Gruberi bursa communicates with the talonavicular joint, the tibiotalar joint, and the EDL tendon sheath, and this needs to be addressed further in future research. In this report, we demonstrated the enhanced MRI features of an inflamed Gruberi bursa, as evidenced by the nature of the pain, fluid distension, and marginal contrast enhancement on post contrast MRI, as well as dramatic clinical improvement after local steroid injection.

## Patient consent statement

Herewith we confirm that a patient consent form has been obtained. For our article entitled: Contrast-enhanced magnetic resonance imaging (MRI) features of Gruberi Bursitis as a very rare cause of dorsolateral ankle pain and swelling: case report and review of the literature
